# Effects of Prenatal Alcohol Exposure on the Volumes of the Lateral and Medial Walls of the Intraparietal Sulcus

**DOI:** 10.3389/fnana.2021.639800

**Published:** 2021-06-07

**Authors:** Marlie Miles, Fleur L. Warton, Ernesta M. Meintjes, Christopher D. Molteno, Joseph L. Jacobson, Sandra W. Jacobson, Christopher M. R. Warton

**Affiliations:** ^1^Department of Human Biology, Faculty of Health Sciences, University of Cape Town, Cape Town, South Africa; ^2^Biomedical Engineering Research Centre, Division of Biomedical Engineering, Faculty of Health Sciences, University of Cape Town, Cape Town, South Africa; ^3^Neuroscience Institute, University of Cape Town, Cape Town, South Africa; ^4^Cape Universities Body Imaging Centre, University of Cape Town, Cape Town, South Africa; ^5^Department of Psychiatry and Mental Health, Faculty of Health Sciences, University of Cape Town, Cape Town, South Africa; ^6^Department of Psychiatry and Behavioral Neurosciences, Wayne State University School of Medicine, Detroit, MI, United States

**Keywords:** intraparietal sulcus (IPS), fetal alcohol spectrum disorder (FASD), arithmetic, magnetic resonance imaging (MRI), cortical volumes

## Abstract

Fetal alcohol spectrum disorders (FASD) continue to be the leading preventable cause of intellectual disability in the U.S., Europe, and in endemic areas, such as the Western Cape region of South Africa. Arithmetic is highly sensitive to prenatal alcohol exposure (PAE). The intraparietal sulcus (IPS) is known to play a critical role in number processing. In this study, we investigate whether smaller IPS volumes play a role in the number-processing deficits observed in children with PAE. Participants were 52 9- to 14-year-old children from a historically disadvantaged community in Cape Town, who are participating in our ongoing studies on the effects of PAE on the brain. The IPS was manually parcellated into its medial (MIPS) and lateral (LIPS) walls on magnetic resonance images. The study aimed to examine: (1) the effects of PAE on IPS regional volumes and asymmetry, (2) whether IPS regional volumes are related to number processing performance and, if so, whether these relations are moderated by PAE and (3) potential mediation by regional IPS volumes of the relation between PAE and number processing performance. Total intracranial volume (TIV) was associated with volumes in all regions except the right LIPS. Both left MIPS and left LIPS volumes were significantly smaller in children in the fetal alcohol syndrome (FAS)/partial FAS (PFAS) group compared to controls. The finding in the left LIPS remained significant after controlling for potential confounders and after adjustment for the smaller overall brain size of the children in the FAS/PFAS group. Smaller left LIPS volumes in the FAS/PFAS group may account for the absence of left-right asymmetry in the LIPS in children with FAS/PFAS compared to controls and nonsyndromal heavily exposed (HE) children. Bilaterally, larger MIPS volumes were associated with better WISC IQ Arithmetic scores. These effects, however, were not moderated by the degree of PAE, and regional IPS volumes did not mediate the effect of PAE on WISC Arithmetic scores. Although we found that certain regions of the IPS were smaller in children with FAS and PFAS, these PAE-induced changes in IPS volume did not mediate the alcohol-related deficits in arithmetic.

## Introduction

Prenatal alcohol exposure (PAE) can lead to a range of structural and functional consequences including delayed growth, atypical facies, and intellectual disability (Jones and Smith, 1973; Hoyme et al., 2005). Fetal alcohol spectrum disorders (FASD) encompass the range of disorders that arise as a result of PAE (Bertrand et al., 2005; Hoyme et al., 2005, 2016). Fetal alcohol syndrome (FAS), the most severe form of FASD, is characterized by a distinctive pattern of craniofacial dysmorphic features (including small palpebral fissures, thin upper lip, and flat philtrum), small head circumference, and delayed pre-and/or postnatal growth, which appears to evolve with age, except in the most severely affected individuals (Jacobson et al., 2021). A diagnosis of partial FAS (PFAS) requires the presence of at least two of the sentinel facial features as well as either small head circumference, delayed growth or neurobehavioral impairment, and confirmed maternal drinking during pregnancy. A large proportion of alcohol-exposed children may, however, lack the characteristic pattern of dysmorphic facial features but exhibit the cognitive and/or behavioral deficits associated with PAE (Hoyme et al., 2005). Our study was conducted in Cape Town, South Africa, where certain historically disadvantaged communities have been reported to have amongst the highest rates of FAS in the world (Croxford and Viljoen, 1999; May et al., 2000, 2013, 2016; Jacobson et al., 2006).

Imaging studies have consistently provided evidence of cortical volume alterations in children prenatally exposed to alcohol (Rajaprakash et al., 2014; Migliorini et al., 2015). In particular, the volume (Archibald et al., 2001; Lebel et al., 2012; Meintjes et al., 2014; Rajaprakash et al., 2014) and function (Woods et al., 2015, 2017; Infante et al., 2017) of the parietal cortex are frequently implicated. Morphometric findings have, however, been inconsistent, with some studies reporting gray matter reductions (Archibald et al., 2001) and others increases (Sowell et al., 2001). Number processing, which is one of the most sensitive neurocognitive deficits associated with PAE (Streissguth et al., 1989, 1994; Kopera-Frye et al., 1996; Burden et al., 2005; Howell et al., 2006; Jacobson et al., 2011a,b), relies on intact functioning of several areas of the parietal cortex. Specifically, visual and verbal processing of quantity are posited to be based in the superior parietal lobule (SPL) and left angular gyrus, respectively, while the quantity system is localized bilaterally in the intraparietal sulcus (IPS; Menon et al., 2000; Naccache and Dehaene, 2001; Dehaene et al., 2003). This area is hypothesized to support number processing irrespective of the notation used.

The IPS is a prominent feature in the parietal lobe that runs from the postcentral sulcus posteriorly and into the occipital lobe, horizontally dividing the superior and inferior parietal lobule (IPL; Clark et al., 2010; Rubin and Safdieh, 2016). Along its route, the IPS is frequently interrupted and has a variable pattern of branching both on the medial and lateral (LIPS) walls (Cunningham, 1890; Zlatkina and Petrides, 2014).

Given the recognized role of the IPS in number sense, we were interested in whether the poorer arithmetic performance observed in children prenatally exposed to alcohol is related to alterations in the structure Sattlerregional. Since automated image segmentation tools to date have not captured patterns of morphological variation in the IPS, nor anatomically defined sub-regions of the IPS, we performed a manual tracing on magnetic resonance images to obtain volumes for the lateral and medial walls of the IPS. The present study examined: (1) the effects of PAE on IPS regional volumes and asymmetry, (2) whether IPS regional volumes are related to number processing performance and, if so, whether these relations are moderated by PAE, and (3) potential mediation by regional IPS volumes of the known relation between PAE and number processing performance.

## Materials and Methods

### Participants

Participants were 52 right-handed, 9- to 14-year-old children from a historically disadvantaged community in Cape Town, South Africa, who are participating in our research on the effects of PAE on brain structure and function, cognition, and behavior (Jacobson et al., 2008; Meintjes et al., 2010; Jacobson et al., 2011c). All are from the same ethnic group. Within this sample, 15 had been diagnosed with FAS or PFAS, 13 were non-syndromal heavily exposed (HE) and 24 were non- or minimally exposed controls. The HE group consists of nonsyndromal children with confirmed heavy PAE who failed to meet the criteria for FAS or PFAS. Due to the small sample sizes, children with FAS or PFAS were grouped together. H.E. Hoyme (HEH), MD, and L.K. Robinson (LKR), MD, two U.S.-based FAS dysmorphologists examined each child at a clinic organized in September 2005 for growth and FAS dysmorphology using the Hoyme et al. (2005) protocol. N. Khaole, MD, a Cape Town-based dysmorphologist, examined the two children who were not able to attend the clinic. Considerable agreement between the examiners was reached on all the dysmorphic features assessed (Jacobson et al., 2008) and a consensus on FAS and PFAS diagnoses was reached during a case conference which included the dysmorphologists and SJ, JJ and CM. The diagnoses have since been confirmed when the children were re-examined by HEH and LKR in 2009 and by a team of dysmorphologists led by HEH in follow-up clinics in 2013 and 2016 (see Jacobson et al., 2021).

Of the 52 participants, 20 were children in our prospective Cape Town Longitudinal Cohort study (Jacobson et al., 2008). The mothers of these children were recruited between July 1999 and January 2002 from an antenatal clinic of a midwife obstetric unit that serves an economically disadvantaged community. Mothers were invited to participate if they averaged at least 1.0 oz absolute alcohol (AA) per day (≈2 standard drinks), or reported at least two incidents of binge drinking (>5 standard drinks/occasion) during the first trimester of pregnancy. Additionally, a random sample of women from the same community initiating antenatal care around the same time was recruited as controls if they drank <0.5 oz AA/day and if they did not binge drink during the first trimester. Mothers were excluded from the study if they were younger than 18 years of age, had a multiple gestation pregnancy, or suffered from chronic illnesses, such as diabetes, epilepsy, or cardiac problems, requiring treatment. Infants were excluded if they presented with major syndromes, neural tube defects, seizures, or if they were part of a multiple-gestation pregnancy or very low birth weight (<1,500 g) or GA <32 weeks. Prospective alcohol exposure data are available for the children from this cohort based on timeline follow-back interviews (Jacobson et al., 2002) conducted with their mothers during pregnancy. The additional 32 participants came from our cross-sectional cohort, who were identified by screening children from an elementary school in a nearby rural section of Cape Town where there is a high level of alcohol use among local farmworkers (Jacobson et al., 2011b). As with the longitudinal sample, two groups of children were recruited: (1) children whose mothers reported heavy drinking during pregnancy, i.e., who consumed at least 14 standard drinks/week (1.0 oz AA/day) on average or who engaged in binge drinking (5 or more drinks/occasion), and (2) controls whose mothers abstained or drank only minimally during pregnancy (≤2 drinks per occasion).

### Procedure

Mothers and children were transported in our research van to our Child Development Research Laboratory at the Faculty of Health Sciences, University of Cape Town (UCT), for neurobehavioral assessment and on the following day to the Cape Universities Brain Imaging Centre (CUBIC) at Tygerberg Hospital for scanning. Each mother completed a written informed consent, and each child provided assent. Breakfast, lunch, and snacks were provided for the mothers and children at each of their visits. The mother received monetary compensation for every visit, and each child received a small gift. Neuropsychological and neuroimaging assessments were administered to each child by examiners who were blind regarding maternal alcohol history and the child’s FASD diagnosis, except for a few severe cases where the FAS diagnosis was apparent. Ethics approval for the study was obtained from the human research ethics committees at Wayne State University and the UCT Faculty of Health Sciences.

### Neuropsychological Assessment

IQ was estimated using 7 of the 10 subtests from the Wechsler Intelligence Scale for Children, Third Edition (WISC-III)—Similarities, Arithmetic, Digit Span, Symbol Search, Coding, Block Design, and Picture Completion—and Matrix Reasoning from the WISC-IV. Sattler’s (1992) formula for computing Short Form IQ was used to estimate the IQ of the children using the aforementioned subtests. Validity coefficients for Sattler Short Form IQ based on five or more subtests consistently exceed *r* = 0.90 (Sattler, 1992). WISC Arithmetic and Digit Span scores were used as a measure of number processing performance. Handedness was assessed on the (Annett, 1970) Behavioral Handedness Inventory.

### Neuroimaging Assessment

All children completed neuroimaging on a 3T Allegra MRI scanner (Siemens Medical Systems, Erlangen, Germany) using the single-channel head coil. High-resolution T1 weighted images of the participants from the longitudinal cohort were acquired in the sagittal plane using a volumetric navigated (Tisdall et al., 2012) multi-echo magnetization prepared rapid gradient echo (MEMPRAGE; Van Der Kouwe et al., 2008) sequence (FOV 256 × 256 × 167 mm^3^, 128 sagittal slices, TR 2,530 ms, TE 1.53/3.21/4.89/6.57 ms, TI 1,100 ms, flip angle 7°, 1.3 × 1.0 × 1.3 mm^3^, 8.07 min). High-resolution anatomical images for the 32 participants of the cross-sectional cohort were also acquired in the sagittal plane using a magnetization prepared rapid gradient echo (MPRAGE) sequence (FOV 256 × 256 × 160 mm^3^, TR 2,300 ms, TE 3.93 ms, TI 1,100 ms, 160 slices, flip angle 12°, 1.3 × 1.0 × 1.0 mm^3^, 6.03 min).

### MRI Analysis

**Figure 1:**
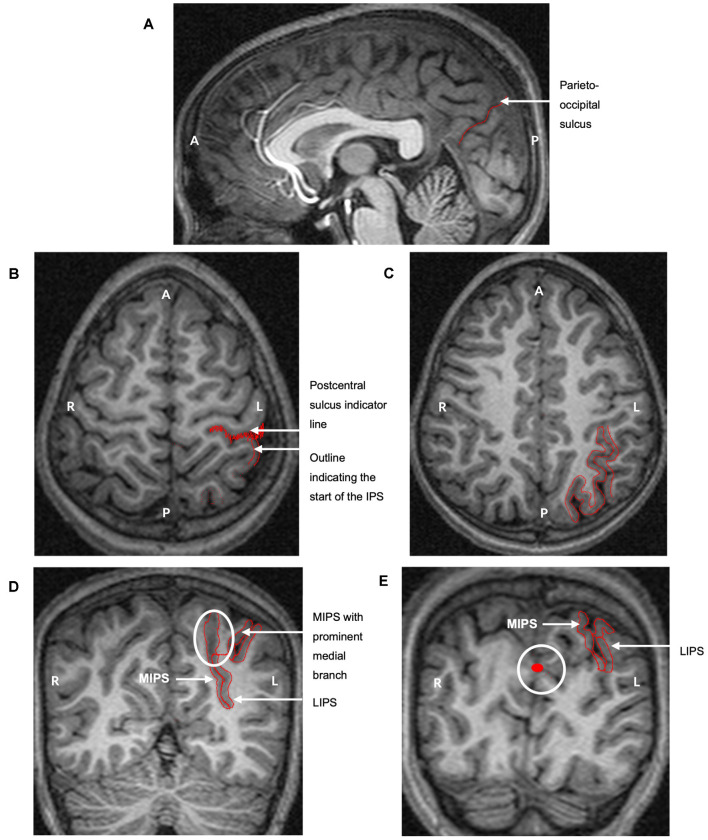
The protocol for manually parcellating the intraparietal sulcus (IPS) is shown on the left hemisphere of a child with fetal alcohol syndrome (FAS)/partial FAS (PFAS). Both the medial (MIPS) and lateral (LIPS) walls of the IPS were traced. **(A)** The parieto-occipital sulcus was first identified on the sagittal plane to indicate the end of the parietal portion of the IPS. Tracings on the transverse planes serve to indicate **(B)** the start of the IPS at the postcentral sulcus and **(C)** the pattern of the sulcus. **(D)** The IPS was finally traced on the coronal sections. The white ellipse indicates the medial branch that was always present near the end of the parietal portion. The thin red line connecting the two sulci is required by the software for the total volume calculation as the area traced must be one continuous structure. **(E)** The white circle indicates tracings from sagittal slices that are visible on the coronal planes, clearly indicating the parieto-occipital border of the IPS (A, Anterior; P, Posterior; L, Left hemisphere; R, Right hemisphere).

The IPS was manually traced on MR images displayed on a Wacom Cintiq tablet using a stylus and MultiTracer software (Woods, 2003). A protocol for manually tracing the cortical volume of the IPS on MR images was developed by CMRW, the senior neuroanatomist at UCT, and MM (Figure 1). All tracings were performed by a single investigator (MM) under the supervision of and in consultation with CW; no computer algorithms were employed to make or edit boundary decisions. A random subset of 10 brains was re-traced by the same investigator to examine intra-rater reliabilities.

The tracing protocol comprised two parts for each hemisphere: (1) tracing all the medial walls of the IPS (MIPS) and (2) tracing the entire sulcus by adding the lateral wall (LIPS). On sagittal and axial planes, we first identified the postcentral and parieto-occipital sulci to delimit the anterior and posterior boundaries of the parietal portion of the IPS (Figures 1A,B,E). The occipital portion was excluded. The IPS was initially traced on the axial sections where the patterns and branches were more clearly visible (Figure 1C). These tracings were then used to guide the tracing on the coronal sections.

MultiTracer software generates volumes for all manually delineated structures. We used “frust” volumes computed from the coronal tracings in each hemisphere of the MIPS and the entire IPS. “Frust” volumes are calculated by assuming that the structure extends from the center of the first plane on which it was drawn to the center of the last plane on which it was drawn with the square root of areas varying linearly when moving from the center of one plane to the center of the next (Woods, 2003). The volume of the LIPS was calculated by subtracting the volume of the MIPS from the total sulcal volume. A prominent medial branch was consistently present posteriorly for the MIPS and was included in the measurements (Figure 1D). Other sulci from the SPL that joined the MIPS in a few cases were excluded. All sulci branching from the LIPS into the inferior parietal lobule (IPL) were kexcluded.

### Statistical Analyses

Statistical analyses were performed using SPSS (version 25, IBM). All variables were normally distributed, except for AA/day which was positively skewed and was log-transformed. The following variables had outliers greater than 3 SD beyond the mean that were recoded to one unit greater than the next highest or lowest value (Winer, 1971): cigarettes/day (*n* = 1), degree of lead exposure (*n* = 1), AA/occasion (*n* = 1) and frequency of drinking days/week (*n* = 2). The alpha level for significance was set at 0.05.

Intra-rater reliabilities were assessed using Pearson correlation and intraclass correlation (ICC) based on a mean-rating two-way mixed-effects model. We report ICC estimates for absolute agreement (Koo and Li, 2016).

Analysis of variance (ANOVA) was used to examine the differences in IPS regional volumes between the FASD diagnostic groups (FAS/PFAS, HE, controls), and between the most severely affected children with FAS or PFAS and controls. Six control variables were considered and assessed as potential confounders: MRI sequence used, child sex and age at scan (years), socioeconomic status (Hollingshead, 2011), maternal cigarette smoking during pregnancy (number of cigarettes smoked/day) and child lead exposure (μg/dl). Using Pearson *r*, we examined the association of each of these control variables with the outcome variables. Any control variable showing even a weak association (at *p* < 0.10) with an outcome variable was controlled for in subsequent analyses with that outcome variable using ANCOVA or multiple regression, respectively. We also present results both before and after adjustment for total intracranial volume (TIV) to examine whether effects of PAE on IPS regional volumes demonstrate a specific vulnerability to the teratogenic effects of PAE or to what degree they are attributable to PAE-related reductions in overall brain size. Additionally, potential effects of PAE on brain asymmetry were examined by comparing regional volumes in left and right hemispheres separately within each diagnostic group.

The relationship between IPS regional volumes and number processing performance was first assessed using Pearson *r*. Subsequently, we examined whether PAE moderates the relation between IPS regional volumes and number processing performance by conducting hierarchical multiple regression analyses. In the first step, the IPS regional volume and PAE were included, and in the second step, the interaction term between the IPS volume and PAE was added to the regression model.

Finally, we performed a series of hierarchical multiple regressions to examine whether IPS regional volumes mediate the effect of PAE on number processing. AA/day during pregnancy was entered in the first step of each regression; the IPS regional volume was entered in the second step. Mediation was inferred if the addition of the IPS volume substantially reduced the magnitude of the regression coefficient for AA/day (Baron and Kenny, 1986). The difference in coefficient tests (Sobel, 1982) was used to assess whether the reduction was statistically significant.

## Results

Sample characteristics are shown in Table 1. There were no between-group differences in terms of child sex and age, maternal education, age, or socioeconomic status. Children in the FAS/PFAS group had smaller TIV and higher blood lead concentrations than controls, and children in both alcohol-exposed groups had lower WISC IQ scores than controls. Although below conventional levels of significance, children in the FAS/PFAS and HE groups performed more poorly on WISC Arithmetic subtest than controls but not on WISC Digit Span. All but two mothers of the control children abstained from drinking: one consumed two drinks on one occasion; the other, two drinks about once/month. Although more of the mothers in the FAS/PFAS and HE groups smoked cigarettes than controls, there were no significant group differences among smokers regarding the number of cigarettes smoked/day, all *p*’s > 0.25. One mother of a child in the FAS/PFAS group reported smoking marijuana about 1 day/month across pregnancy. None of the other mothers reported using marijuana, and none reported using cocaine, methaqualone, or any other illicit drugs.

**Table 1:** Sample characteristics.

	Alcohol exposed					
	FAS/PFAS	HE	Controls	Total	*F* or χ^2^	*p*
	(*n* = 15)	(*n* = 13)	(*n* = 24)	(*N* = 52)		
**Child**						
Sex: *n* = male (%)	8 (15.4)	6 (11.5)	12 (23.1)	26 (50.0)	0.14	0.93
Age (years) at scan	10.9 (1.1)	11.4 (0.9)	11.2 (1.1)	11.2 (1.1)	0.62	0.54
Total intracranial volume (×10^6^mm^3^)(TIV)^a^	1.29 (0.17)	1.43 (0.15)	1.53 (0.14)	1.43 (0.18)	10.95	<**0.001**
Blood lead concentration (μg/dl)^b^	10.4 (6.3)	7.9 (4.0)	6.4 (2.1)	7.9 (4.4)	4.37	**0.02**
WISC IQ^c^	64.5 (8.4)	65.3 (10.9)	75.6 (1.0)	69.8 (11.0)	7.74	**0.001**
WISC Arithmetic	6.0 (2.4)	6.0 (2.6)	7.9 (3.1)	6.9 (2.9)	2.94	0.06
WISC Digit Span	7.1 (3.2)	7.2 (2.4)	7.9 (2.6)	7.9 (2.6)	0.47	0.63
**Maternal**						
Education (years)^d^	7.9 (3.0)	8.1 (2.1)	8.9 (1.8)	8.4 (2.3)	1.25	0.30
Mother’s age at delivery (years)	28.1 (7.8)	25.5 (5.1)	27.4 (4.7)	27.2 (5.8)	0.74	0.48
Socioeconomic status (SES)^e^	17.1 (6.89)	19.7 (10.38)	20.8 (8.86)	19.5 (8.73)	0.80	0.45
**Substance use**^f^						
Absolute alcohol/day (oz)^g^	2.1 (2.7)	1.6 (1.3)	0.002 (0.01)	0.1 (1.8)	8.85	**0.001**
Absolute alcohol/occasion (oz)^h^	4.5 (2.3)	5.4 (3.7)	0.1 (0.3)	2.7 (3.3)	31.47	<**0.001**
Frequency (days/week)^i^	2.5 (2.0)	1.9 (1.0)	0.01 (0.1)	1.2 (1.6)	26.15	<**0.001**
Cigarettes users, *n*(%)	14 (93.3)	13 (100.0)	9 (37.5)	36 (69.2)	21.21	<**0.001**
Cigarettes/day^j^	8.9 (5.4)	10.6 (8.0)	9.9 (11.7)	9.8 (8.0)	0.15	0.86

*All variables are Mean (SD). FAS, fetal alcohol syndrome; PFAS, partial FAS; HE, nonsyndromal heavy alcohol exposed; WISC, Wechsler Intelligence Scale for Children; 1 oz absolute alcohol ≈2 standard drinks; ^a^FAS/PFAS < Controls (p < 0.001); ^b^FAS/PFAS > Controls (p = 0.013); ^c^FAS/PFAS < Controls (p = 0.004); HE < Controls (p = 0.01); ^d^Of N = 52, 48 primary caregivers were the biological mothers of the children; 2 (HE) were the fathers, 1 (Control) was the grandmother, and 1 (FAS/PFAS) was another relative; ^e^Hollingshead (2011) Four Factor Index of Socioeconomic Scale; ^f^One mother of a child with FAS used marijuana 0.87 days/month during pregnancy; ^g^FAS/PFAS > Controls (p = 0.001), HE > Controls (p = 0.016); ^h^FAS/PFAS, HE > Controls (p < 0.001); ^i^FAS/PFAS, HE > Controls (p < 0.001); ^j^Smokers only. Bold denotes significance at *p* < 0.05*.

Intra-rater reliabilities are presented in Table 2. As per the convention proposed by Koo and Li (2016), volumes of all regions demonstrated good reliabilities (ICC estimates between 0.75 and 0.9), except for the right LIPS where it was moderate (ICC estimate between 0.5 and 0.75).

**Table 2:** Intra-rater reliability of the ROI volumes for 10 randomly selected participants.

	Pearson	ICC (absolute agreement)	95% CI
	*r*(*p*)	*r*(*p*)	
Left MIPS	0.774 **(0.009)**	0.757 **(0.004)**	0.286–0.934
Right MIPS	0.906 **(<0.001)**	0.850 **(<0.001)**	0.449–0.962
Left LIPS	0.809 **(0.005)**	0.821 **(0.001)**	0.426–0.953
Right LIPS	0.696 **(0.025)**	0.694 **(0.01)**	0.163–0.914

*MIPS, Medial wall; LIPS, Lateral wall, ICC, Intraclass correlation coefficient. Bold denotes significance at *p* < 0.05*.

Table 3 shows the associations of volumes in each region with TIV and with potential confounders. Imaging sequence, child sex, and smoking each showed an association with one ROI volume. By contrast, TIV was related to three of the four ROI volumes. Although lead exposure levels in children in the FAS/PFAS group were higher than in either of the other two groups, it showed no association with any of the volumes examined here and was, therefore, not controlled for in subsequent analyses.

**Table 3:** Associations of volumes with TIV and potential confounders (*N* = 52).

	Total intracranial volume		Imaging^1^		Age at scan		Sex of child^1^		Socio-economic status		Cigarettes/day^2^		Lead exposure^2^	
	*r*	*p*	*r*	*p*	*r*	*p*	*r*	*p*	*r*	*p*	*r*	*p*	*r*	*p*
Left MIPS	0.38	**0.005**	0.11	0.46	−0.18	0.20	0.01	0.92	0.04	0.77	−0.24	**0.08**	−0.17	0.23
Right MIPS	0.27	**0.05**	0.08	0.56	−0.16	0.25	−0.00	0.98	−0.11	0.44	0.02	0.88	−0.05	0.73
Left LIPS	0.26	**0.06**	0.31	**0.03**	−0.05	0.72	0.25	**0.07**	−0.07	0.61	−0.18	0.20	−0.22	0.11
Right LIPS	0.22	0.11	0.22	0.11	−0.15	0.30	0.08	0.58	−0.05	0.74	0.04	0.80	−0.05	0.73

*Values are Pearson correlation coefficients. ^1^For categorical variables, point biserial correlation was used. ^2^One outlier (>3 SD beyond the mean) recoded to 1 unit higher than the next highest value. Bold denotes association at p < 0.10*. MIPS, Medial wall; LIPS, Lateral wall; TIV, total intracranial volume.

**Figure 2:**
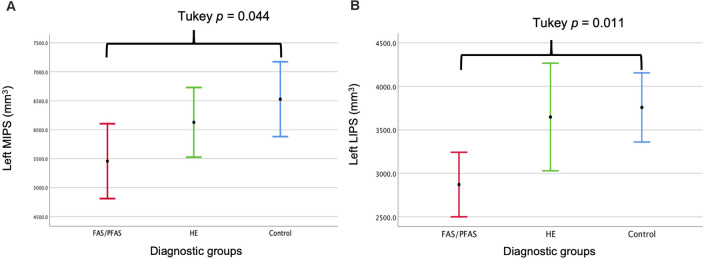
Comparison of the **(A)** left medial (M) and **(B)** left lateral (L) IPS volumes by diagnostic group (mean ± 95% Confidence Intervals).

As shown in Table 4 and Figure 2, there was a significant association between the FASD group and left LIPS volumes, even after control for potential confounders. Adjustment for TIV reduced the association between group and left LIPS volume to below conventional levels of significance, suggesting that the effect reflected, in part, smaller overall brain volume in the FAS/PFAS group compared to controls. Direct comparison of the most severely affected children in the FAS/PFAS group and controls revealed that the left LIPS volumes were significantly smaller in children with FAS/PFAS than in controls (*t* = 3.17, *p* = 0.003), even after adjustment for potential confounders (*F* = 6.16, *p* = 0.02), TIV (*F* = 5.52, *p* = 0.02), or both (*F* = 4.48, *p* = 0.04). Although the association between FASD diagnosis and left MIPS volumes just missed conventional levels of significance (*p* = 0.06) and was no longer even a trend after inclusion of the potential confounders or adjustment for TIV (Table 4), direct comparison of the most severely affected children in the FAS/PFAS and control groups indicated that left MIPS was significantly smaller for the FAS/PFAS group than for controls (*t* = 2.32, *p* = 0.03) but that this finding does not survive after control for smoking (*F* = 2.54, *p* = 0.12) or TIV (*F* = 1.35, *p* = 0.25). Volumes of the right MIPS and right LIPS did not differ between children in the FAS/PFAS and control groups (*p* = 0.22 and *p* = 0.09, respectively).

**Table 4:** Comparison of volumes by FASD diagnosis (*N* = 52).

	FAS/PFAS (*n* =15)	HE (*n* = 13)	Controls (*n* =24)						
	Mean (SD)	Mean (SD)	Mean (SD)	*F*	*p*	*F*^1^	*p*^1^	*F*^2^	*p*^2^
Left MIPS^a^	5,458 (1,169)	6,128 (995)	6,528 (1,530)	3.05	0.06	2.21	0.12	0.60	0.55
Right MIPS	5,800 (941)	5,645 (2,016)	6,303 (1,369)	1.05	0.36	1.05	0.36	0.47	0.63
Left LIPS^b,c^	2,871 (670)	3,648 (1,023)	3,758 (941)	4.85	0.01*	3.95	**0.03**	2.90	0.07
Right LIPS	2,668 (726)	2,915 (1,038)	3,211 (1,072)	1.46	0.24	1.46	0.24	0.47	0.62

^1^ANCOVA controlling for potential confounders; FAS, fetal alcohol syndrome; PFAS, partial FAS; HE, nonsyndromal heavy alcohol exposed; FASD, fetal alcohol spectrum disorders. ^2^ANCOVA controlling for TIV. Potential confounders are ^a^Cigarettes/day, ^b^Imaging sequence, ^c^Sex of the child. *Tukey *post hocs*: Left LIPS: FAS/PFAS < Controls (*p* = 0.01). MIPS, Medial wall; LIPS, Lateral wall. Bold denotes significance at *p* < 0.05.

While no left-right asymmetry was evident in the medial wall of the IPS in any of the groups, asymmetry in the lateral IPS was seen in control children that were not evident in children with FAS/PFAS, and evident below conventional levels of significance in the HE group (Table 5).

**Table 5:** Comparison of hemispheric asymmetry by FASD diagnosis (*N* = 52).

	FAS/PFAS (*n* =15)			HE (*n* =13)			Controls (*n* =24)		
	Mean (SD)	*t*	*p*	Mean (SD)	*t*	*p*	Mean (SD)	*t*	*p*
Left MIPS	5,458 (1,168)	−1.45	0.17	6,128 (995)	1.22	0.25	6,528 (1,530)	0.95	0.35
Right MIPS	5,800 (941)			5,645 (2,016)			6,303 (1,369)
Left LIPS	2,871 (670)	1.12	0.28	3,648 (1,023)	2.10	0.06	3,758 (941)	2.53	**0.02**
Right LIPS	2,668 (726)			2,915 (1,038)			3,211 (1,072)		

*FAS, fetal alcohol syndrome; PFAS, partial FAS; HE, nonsyndromal heavy alcohol exposed. MIPS, Medial wall; LIPS, Lateral wall. Bold denotes significance at *p* < 0.05*.

**Figure 3:**
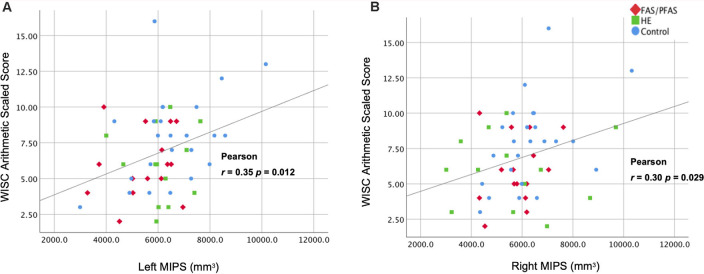
Associations of WISC-IV Arithmetic scores with **(A)** left and **(B)** right medial IPS (MIPS) volumes.

**Figure 4:**
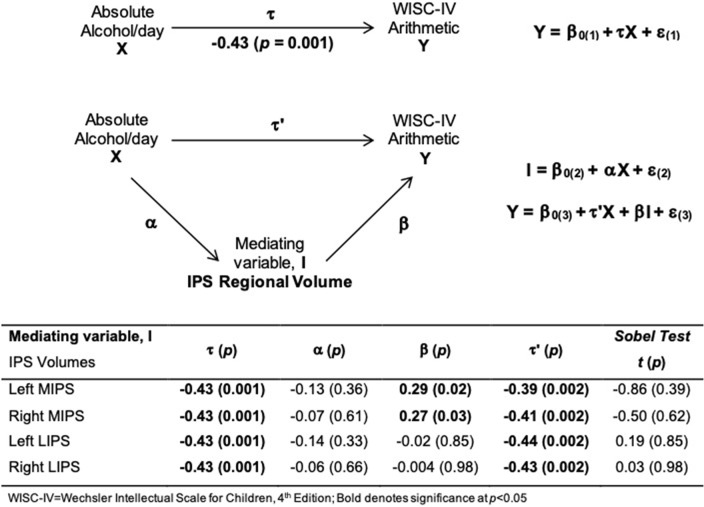
Path model illustrating mediation of the effect of alcohol consumption on WISC-IV Arithmetic by smaller IPS volumes.

Larger left and right MIPS were associated with higher WISC Arithmetic scores (Table 6, Figures 3A,B). While multiple regression showed that both MIPS volumes and PAE affect WISC Arithmetic performance, PAE did not moderate the relations between MIPS volumes and WISC Arithmetic. Regional IPS volumes also did not mediate the effect of PAE on WISC Arithmetic (Figure 4).

**Table 6:** Relation between math performance on the WISC Arithmetic and regional IPS volumes, before and after adjustment for degree of prenatal alcohol exposure and potential moderation by prenatal alcohol exposure of the relations between regional IPS volumes and WISC Arithmetic.

		**Model 1**		**Model 2**		
	*r* (*p*)	*β*^1^ (*p*)	*β*^2^ (*p*)	*β*^1^′ (*p*)	*β*^2^′ (*p*)	*β*^3^ (*p*)
Left MIPS	**0.35 (0.01)**	**0.29 (0.02)**	−**0.39 (0.002)**	**0.28 (0.03)**	−**0.40 (0.002)**	−0.09 (0.46)
Right MIPS	**0.30 (0.03)**	**0.27 (0.03)**	−**0.41 (0.002)**	**0.27 (0.03)**	−**0.42 (0.001)**	−0.17 (0.18)
Left LIPS	0.04 (0.81)	−0.02 (0.85)	−**0.44 (0.002)**	−0.03 (0.85)	−**0.44 (0.002)**	0.004 (0.98)
Right LIPS	0.02 (0.87)	−0.004 (0.98)	−**0.43 (0.002)**	0.004 (0.98)	−**0.43 (0.002)**	0.02 (0.88)

r, Pearson correlation coefficient; Model 1: WISC Arithmetic = *β^0^* + *β^1^*(ROI Volume)+ *β^2^*(AA/day); Model 2: WISC Arithmetic = *β^0^* + *β^1^′*(ROI Volume)+*β^2^′*(AA/day) + *β^3^*(ROI volume*AA/day). MIPS, Medial wall; LIPS, Lateral wall; WISC, Wechsler Intelligence Scale for Children. Bold denotes significance at *p* < 0.05.

## Discussion

In this study we manually traced the IPS on MR images to investigate the effects of PAE on regional IPS volumes and whether alterations in IPS volume play a role in number processing deficits observed in PAE (Meintjes et al., 2010; Woods et al., 2015). While both the left lateral and medial walls were smaller in children in the FAS/PFAS group compared to controls, group differences in the medial wall did not survive after control for smoking or TIV. Notably, the asymmetric larger volumes of the left LIPS compared to the right seen in control children were not evident in children with FAS/PFAS, which may account for volume reductions seen in these children in this region. Although bilaterally larger medial walls were associated with better performance on the WISC Arithmetic test—effects that were not moderated by PAE—regional IPS volumes did not mediate the effect of PAE on WISC Arithmetic.

Although numerous studies have examined the effects of PAE on brain volumes (see Lebel et al., 2011 review), none have focused specifically on the IPS. Our results extend findings from Archibald et al. (2001) of parietal lobe reductions in 11- to 13-year-old children with FAS to a specific structure. Similar to Sowell et al. (2001), who found structural abnormalities in PAE individuals aged 8–22 years predominantly in the *left* posterior temporo-parietal cortices, we also found *only* the left IPS to be smaller in children in the FAS/PFAS group. Although both the medial and lateral walls were smaller, reductions in the medial wall did not survive after controlling for maternal smoking during pregnancy. Prenatal exposure to smoking has been associated with reduced brain volumes of the frontal lobe and cerebellum (Ekblad et al., 2010), as well as increased activity in the left SPL during tasks involving working memory (Longo et al., 2014). Alternatively, compared with alcohol recall, smoking is more habitual and, therefore, easier to recall (Jacobson et al., 2002). It is also more readily quantifiable than alcohol and may, therefore, account for more of the shared variance between these exposures and the outcome. Thus, analyses controlling for alcohol may overattribute deficits to smoking and under control for the effect of alcohol exposure.

Lesion and imaging studies have confirmed the role of the IPS in arithmetic and number processing (Cohen and Dehaene, 1995; Menon et al., 2000; Isaacs et al., 2001; Naccache and Dehaene, 2001; Dehaene et al., 2003). Localizing the exact areas within the IPS involved in performing arithmetic tasks could aid in better understanding the impact of the PAE-related structural alterations on mathematical abilities and potentially developing remedial or pharmacological treatments. In our study, both the left- and right MIPS volumes but not the lateral walls were associated with WISC Arithmetic scores. Associations remained essentially unchanged after adjustment for the degree of alcohol exposure and potential interaction effects. Five key parietal areas for number processing have been suggested, including the left and right posterior SPL and the IPS, with its anterior portion being most active during the interpretation of magnitude (Dehaene et al., 2003). The location of the posterior SPL delineated by Dehaene et al. (2003) and used by Woods et al. (2015) in fMRI studies of number processing corresponds neuroanatomically with the medial branch of the MIPS in the current study. The posterior SPL is activated during certain arithmetic calculations, such as counting (Piazza et al., 2003) and number comparison (Pinel et al., 2001). Since the posterior SPL and anterior portion of the IPS comprise most of our MIPS volumes, it is not surprising that children with larger MIPS volumes performed better on the WISC arithmetic assessments.

During the process of manual tracing, extreme inter-subject variations were observed in the branching patterns and shapes of the sulcus between hemispheres. Interhemispheric asymmetry, in terms of volume, observed in the LIPS in controls and HE children were not seen in children in the FAS/PFAS group or in the MIPS in any of the FASD diagnostic groups. Structural asymmetry is known to be typical in healthy brains (Watkins et al., 2001). Although PAE-related changes in typical asymmetry have been reported previously (Sowell et al., 2002), this is the first study to assess the asymmetry of the IPS.

A limitation in this study was the small sizes of the FAS/PFAS and HE groups. Thus, the results would need to be confirmed in a larger independent study. Two MRI sequences (MPRAGE for *n* = 32; and MEMPRAGE for *n* = 20) were used for scanning, which may have altered the MR images slightly and could have affected the volume results. This was, however, adjusted for by including imaging sequence as a potential confounder. Since the children in our cohort were recruited from an educationally and socio-economically deprived community, our findings need to be examined in children raised in less deprived environments to see if these findings also hold for them. Strengths of the current study include the use of the timeline follow-back approach, which allowed the researchers to examine dose-dependent effects, and manual tracing, which allowed an expert neuroanatomist to make informed decisions related to branching and structural variations observed in the IPS.

This study used manual tracing on MR images to examine the effects of PAE on IPS volumes and associations with number processing. In contrast to other studies that examined volumes of the parietal lobe as a whole, our study examined the volumes of sub-regions within the IPS specifically. The left IPS was found to be smaller in children with FAS or PFAS, which fell below conventional levels of significance, after adjustment for overall reductions in brain size. This volume reduction may account for the loss of asymmetry seen in children with FAS and PFAS in the lateral wall. Bilaterally, increasing MIPS volumes were associated with better arithmetic performance on the WISC IQ test.

## Data Availability Statement

The raw data supporting the conclusions of this article will be made available by the authors, without undue reservation.

## Ethics Statement

The studies involving human participants were reviewed and approved by the University of Cape Town Faculty of Health Sciences Human Research Ethics Committee and the Wayne State University Institutional Review Board. Written informed consent to participate in this study was provided by the participants’ legal guardian/next of kin.

## Author Contributions

MM together with CW designed the protocol for tracing the areas from which the volumes were calculated, and MM completed the tracings for all the subjects. She also conducted the review of the literature, statistical analyses, interpretation, and write-up of the findings. EM, FW, and CW provided supervision for MM’s project and collaborated on the write-up, study design, and statistical analyses. SJ, JJ, and EM designed, implemented, oversaw the data collection on the Cape Town FASD study, collaborated on the statistical analyses, write-up, and interpretation of the findings from this study. Maternal interviews were administered by CM.

## Conflict of Interest

The authors declare that the research was conducted in the absence of any commercial or financial relationships that could be construed as a potential conflict of interest.
